# Effects of alcohol-related problems on the costs of frequent emergency department use: an economic analysis of a case–control study in Spain

**DOI:** 10.3389/fpubh.2024.1322327

**Published:** 2024-12-03

**Authors:** Clara Oliveras, Pol Bruguera, Imanol Cordero-Torres, Andrea Millán-Hernández, Maria Teresa Pons-Cabrera, Pablo Rodrigo Guzmán Cortez, Marta Gómez-Ramiro, Mireia Vázquez, Roger Borràs, Maria Asenjo-Romero, Eduard Vieta, Antoni Gual, Hugo López-Pelayo, Mercè Balcells-Oliveró

**Affiliations:** ^1^Addictions Unit, Psychiatry and Psychology Service, ICN, Hospital Clinic Barcelona, Barcelona, Spain; ^2^Department of Psychiatry and Clinical Psychology, Hospital Clínic de Barcelona, Barcelona, Spain; ^3^Health and Addictions Research Group, Institut d’Investigacions Biomèdiques August Pi i Sunyer (IDIBAPS), Barcelona, Spain; ^4^Red de Investigación en Atención Primaria de Adicciones (RIAPAd), Madrid, Spain; ^5^Emergency Department, Hospital Clínic de Barcelona, Barcelona, Spain; ^6^Centro de Investigación en Red de Salud Mental (CIBERSAM), Madrid, Spain; ^7^Barcelona Clínic Schizophrenia Unit, Institute of Neuroscience, Hospital Clínic, University of Barcelona, IDIBAPS, Barcelona, Spain; ^8^Psychiatry Service, Complejo Hospitalario Universitario de Vigo, SERGAS, Translational Neuroscience Research Group, Galicia Sur Health Research Institute (IISGS), Vigo, Spain; ^9^Institute of Neuroscience and Medical Statistics Core Facility, Hospital Clínic de Barcelona, Barcelona, Spain; ^10^Bipolar and Depressive Disorders Unit, Institute of Neuroscience, Hospital Clínic, University of Barcelona, IDIBAPS, Barcelona, Spain

**Keywords:** healthcare costs, alcohol, psychiatry, emergency department, frequent users

## Abstract

**Introduction:**

Alcohol-related problems increase the probability of frequent emergency department (ED) use. In this study, we compared the direct healthcare expenses incurred during a single visit among frequent and non-frequent ED users and analyzed the impact of alcohol-related issues in healthcare costs arising from ED usage.

**Methods:**

The study relied on secondary analyses of economic data from a 1:1 matched case–control study with the primary aim of identifying the clinical characteristics of hospital ED frequent users in a Mediterranean European environment with a public, universal, and tax-funded health system. The participants ranged in age from 18 to 65 years and underwent ED visits at a high-complexity Spanish hospital (cases ≥5 times, controls <5) from December 2018 to November 2019. Each case was matched to a control with the same age, gender, and date of attendance at the ED. Clinical data and direct healthcare costs for a single ED visit were obtained by a retrospective review of the first electronic medical register. Costs and duration of stay were compared between cases and controls using paired-samples *t*-tests, and ED users with and without alcohol-related problems were compared using bivariate (independent-samples *t*-tests, one-way analysis of variance, Chi square tests, and multiple linear regression) and multivariate analyses (multiple linear regression models with backward stepwise selection algorithm, and dependent variable: total mean direct costs).

**Results:**

Among 609 case–control pairs (total *n* = 1218), mean total healthcare direct costs per ED visit were 22.2% higher among frequent compared with non-frequent users [mean difference 44.44 euros; 95% confidence interval (CI) 13.4–75.5; t(608) = 2.811; *p* = 0.005]. Multiple linear regression identified length of stay, triage level, ambulance arrival, and the specialty discharging the patient as associated with total healthcare costs for frequent users. In bivariate analyses, a history of alcohol-related problems was associated with a 32.5% higher mean total healthcare costs among frequent users [mean difference 72.61 euros; 95% confidence interval 25.24–119.97; t(320.016) = 3.015; *p* = 0.003].

**Conclusion:**

The findings confirm the high cost of frequent ED use among people with alcohol-related problems, suggesting that costs could be reduced through implementation of intervention protocols.

## 1 Introduction

Most patients use emergency departments (EDs) sporadically for isolated pathologies, but some patients use these services frequently, representing a disproportionate amount of healthcare costs (1). Although definitions of frequent ED use vary (2), a common definition is five or more visits annually (3). In high-income countries, the percentage of frequent users (FUs) of ED is between 0.3 and 8% of all patients who present to emergency services, representing up to 28% of all ED visits (4). FUs are not only heavy users of acute services, but they also frequently use other health services (5), which suggests that they are sicker and more vulnerable. This is supported by a higher-than-expected mortality (6). These factors are likely to increase healthcare system costs.

Drug use-related disorders and other psychiatric diseases seem to increase the probability of frequent ED use (7, 8), which is especially true for alcohol addiction (9).

From 1990 to 2017, there was a global increase in individual alcohol consumption, prevalence of current drinkers, and proportion of episodic heavy drinkers among adults, whereas the proportion of lifetime abstinence declined. These trends are anticipated to continue in the coming years (10). Patients with alcohol-related problems (ARPs) are less likely to use primary care services than the rest of the population (11), but they are more likely to use emergency services (12). Furthermore, alcohol-related ED visits seem to be increasing in frequency, duration, and resource consumption (13). For instance, patients with emergency department visits related to drug use are more likely to receive diagnostic tests, such as toxicology screenings (14).

As described for other psychiatric illnesses (15), ARPs predict higher costs, not only in the ED (16), but also in the entire healthcare network (17).

Currently, the majority of studies describing the characteristics of FUs have been conducted in English-speaking countries, such as the United States, Canada, Australia, or the United Kingdom (18–20), with fewer studies available from other regions (21). Globally, healthcare costs associated with frequent ED use have received less attention (2).

This manuscript presents the secondary analyses of economic data obtained from a case–control study with the primary objective of outlining the clinical characteristics of hospital ED FUs in a Mediterranean European country (Spain). The results of that study indicated that a history of ARPs [adjusted odds ratio = 1.82 (95% confidence interval [CI] 1.26–2.64), *p* = 0.001] increased the probability of frequent utilization of emergency services (22). The main aims of the secondary analyses presented in this manuscript were to compare direct healthcare costs of a single urgent visit between frequent and non-frequent users of hospital emergency services and to explore the role of ARPs in direct healthcare costs of frequent ED use in a universal, public, tax-financed national health system. A secondary objective was to investigate the factors influencing the direct costs of ED utilization among individuals who are non-frequent users.

## 2 Methods

### 2.1 Study design and setting

A retrospective matched case–control study was conducted to characterize the clinical profile of ED FUs at a tertiary hospital located in a metropolitan city (Barcelona) in Spain.

The ED is responsible for treating internal medicine, psychiatric, trauma, and surgical emergencies. Electronic health records are used to track all healthcare encounters within the center and provide access to clinical care data. The ED is located in a specific building of the hospital, and different levels of acute care are assigned to different floors. The Spanish healthcare system is public, universal, and free of charge (tax-financed; Beveridge model (23)).

The study adhered to the STROBE (Strengthening the Reporting of Observational Studies in Epidemiology) Statement Checklist for case–control studies (24) (Supplementary material 1).

The main objective of the study was to determine the significance of alcohol-related issues in the frequent utilization of an ED at a general hospital in a European Mediterranean society with a public, universal, tax-funded healthcare system (Spain). Another objective was to investigate the influence of other drug use-related disorders on frequent ED use in this environment. The hypothesis for the main study was that a history of alcohol-related problems and other drug use-related disorders would increase the probability of frequent attendance at the ED.

In this article, the secondary analyses of economic data from the aforementioned study (22) are reported.

The local Ethics Committee for Clinical Research at the Hospital Clinic of Barcelona has granted ethical approval (HCB/2019/0717). The investigation was conducted in accordance with the guidelines on Good Clinical Practice (CPMP/ICH/135/95) and with the ethical principles stated in the Declaration of Helsinki 1964, as revised at the 64th World Medical Association General Assembly held in Fortaleza, Brazil, in October 2013.

### 2.2 Participant selection

All cases (FUs) were all adults (ages 18 to 65 years) who had at least five visits to the hospital ED from 1 December 2018 to 30 November 2019. Each case was matched by age, gender, and date of ED attendance to one control (a non-frequent user with <5 yearly ED visits during that period).

During the study period, the ED received 103,668 visits from 75,410 patients. As in previous studies (4), the initial ED attendance recorded in the electronic register from 1 December 2018 to 30 November 2019 was utilized to match, by date of presentation to the ED, each case with a control of the same age and gender, extract clinical characteristics, and calculate direct healthcare costs per urgent visit.

**Figure 1:**
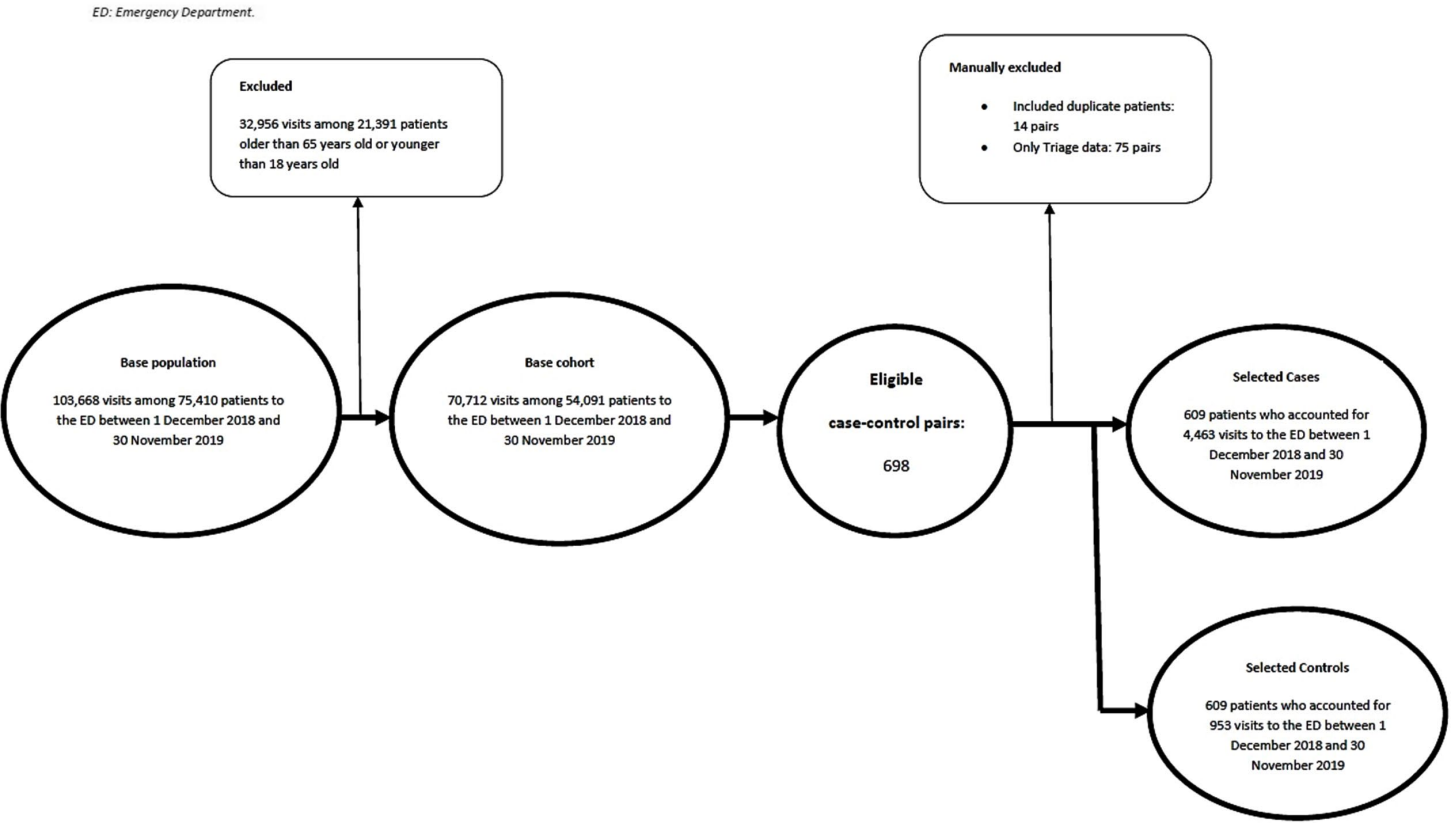
Flowchart of participant selection.

After electronically applying inclusion and exclusion criteria, we have identified 698 case–control pairs. Of these, 89 pairs were manually excluded, including 14 instances of duplicate cases and 75 instances of absence of a medical note. A final list of 609 case–control pairs was generated, giving a total sample size of 1218 (Figure 1).

Before data extraction started, sample size calculations were performed based on estimates from previous studies (25). Assuming that 12% of controls would have a history of ARP, to detect a minimum odds ratio of 1.6 for emergency department frequent use with a power of 80% and a type I error of 0.5, a minimum of 567 case–control pairs with one matched control per case was needed, for a total sample size of 1158.

### 2.3 Methods of measurement

The variables obtained from each participant in the main case–control study are the following:

Outcome: frequent use of ED (≥5 visits to the ED from 1 December 2018 to 30 November 2019).

Exposures of interest include the history of any alcohol-related issues, as well as reports of other drug use-related disorders.

Covariates: age, gender, residence near the hospital, number of visits to the ED during a year, length of stay in the ED per visit (in minutes), night admission to the ED, ambulance arrival, report of other psychiatric comorbidity, report of organic comorbidity, report of alcohol drinking pattern, psychiatric assessment in the ED, social assessment in the ED, assessment by a non-psychiatric medical specialty in the ED, triage level at admission to the ED, type of specialty that discharged the patient from the ED, month of attendance at the ED, time of day at admission, type of psychiatric comorbidity, type of other drug use, situation at discharge.

To elaborate on a “Yes” in the variable “history of any alcohol-related problem,” the medical report had to include the history of diagnoses according to the International Classification of Diseases, Tenth Revision (ICD-10) (26) and the Diagnostic and Statistical Manual of Mental Disorders, Fifth Edition (DSM-5) (27), given in the table included in Supplementary material 2. The presence in the medical report of clinical presentations compatible with these pathologies, even if they were not coded through a standardized disease classification system, was also considered affirmative for the presence of ARPs. The reporting of other clinical conditions, such as accidents and trauma, when clearly related to alcohol use was also included as ARPs.

The following variables were automatically provided by the electronic health record system (SAP^®^ software): age, gender, triage level at admission to the ED, month of attendance at the ED, time of day at admission, and length of stay (LoS). For other variables, in accordance with the general recommendations for this particular methodology (28), three team members conducted a chart data extraction. Two are psychiatrists specialized in addictions (CO and MTPC), and one is a nurse specialized in mental health (AMH). Prior to the commencement of extraction, all abstractors attended a meeting with the corresponding author (CO) to train in chart review methodology. If a condition was not mentioned, the abstractors documented that the condition was not present. Before formally beginning the extraction process, they reviewed 20 sample cases to assess reliability. This was confirmed by a Fleiss multi-rater Kappa statistic >0.6 for all variables. Throughout the extraction period, chart abstractors and senior researchers (AG, MB-O, HL-P) regularly met to resolve disputes and review coding rules.

Further details on the data extraction criteria are described elsewhere (22).

For the secondary analyses presented in this article, financial data on direct medical costs (in euros, €) per ED visit were obtained from the hospital’s financial department (Supplementary material 3).

### 2.4 Data analysis

The statistical analyses were performed using SPSS version 23.0 (IBM Corp., Armonk, NY, United States) and Stata 14 (StataCorp LLC, College Station, TX, United States). Data were summarized with descriptive analyses: continuous quantitative variables with total numbers, mean (M), and standard deviation (SD) and categorical variables with counts and percentages. To compare the costs and LoS between cases and controls, Student’s *t*-tests for paired samples were used.

Subsequently, the subgroups of frequent and non-frequent users were analyzed separately to ascertain which patient characteristics and professional interventions influence direct total costs per ED visit within each subgroup. ED users with ARPs were compared to users without ARPs using bivariate analyses (independent-samples *t*-tests, one-way analysis of variance, Chi-square tests, and multiple linear regression). In multivariate analyses, multiple linear regression models were used with total direct costs per ED visit as the dependent variable. Variables deemed clinically relevant and that were statistically significant (*p* < 0.05) for the dependent variable in bivariate analyses were incorporated into adjusted models employing a backward stepwise selection algorithm.

## 3 Results

### 3.1 Descriptive analyses

The sample included 609 case–control pairs (*N* = 1218; Tables 1–3). During the one-year period (1 December 2018 to 30 November 2019), controls accounted for 953 visits to the ED, whereas FUs accounted for 4,463 visits. The FUs visited the ED a mean of 7.34 (SD = 4.16) times per year (minimum of 5 and maximum of 42; 88.8% visited the ED ≤10 times during the study period). Controls presented a mean of 1.56 (SD = 0.87) times per year to the ED. The mean total direct expenses per ED visit were 244.87 € (SD = 268.86) among ED FUs and 200.43€ (SD = 310.12) among controls. There were no missing data.

**Table 1:** Patient characteristics (clinical and sociodemographic data) of ED FUs (cases) vs. non-frequent users (controls): descriptive statistics.

	ED FUs (*n* = 609)	ED FUs with ARPs (*n* = 182)	ED FUs without ARPs (*n* = 427)	Controls (*n* = 609)	Controls with ARPs (*n* = 86)	Controls without ARPs (*n* = 523)
Age (years)	M = 44.57 SD = 13.7	M = 46.84 SD = 12.236	M = 44.51 SD = 13.840	M = 44.57 SD = 13.7	M = 47.92 SD = 12.69	M = 44.02 SD = 13.50
Gender (Male)	346 (56.8%)	136 (74.7%)	210 (49.2%)	346 (56.8%)	57 (66.3%)	289 (55.3%)
Number of visits to the ED during a year	M = 7.34 SD = 4.16	M = 8.31 SD = 4.98	M = 6.92 SD = 3.69	M = 1.56 SD = 0.87	M = 1.57 SD = 0.902	M = 1.56 SD = 0.869
Month of Attendance at ED	January 102 (16.7%)	January 33 (18.1%)	January 69 (16.2%)	January 102 (16.7%)	January 18 (20.9%)	January 84 (16.1%)
	February 74 (12.2%)	February 28 (15.4%)	February 46 (10.8%)	February 74 (12.2%)	February 7 (8.1%)	February 67 (12.8%)
	March 71 (11.7%)	March 18 (9.9%)	March 53 (12.4%)	March 71 (11.7%)	March 5 (5.8%)	March 66 (12.6%)
	April 69 (11.3%)	April 19 (10.4%)	April 50 (11.7%)	April 69 (11.3%)	April 5 (5.8%)	April 64 (12.2%)
	May 39 (6.4%)	May 13 (7.1%)	May 26 (6.1%)	May 39 (6.4%)	May 6 (7.0%)	May 33 (6.3%)
	June 43 (7.1%)	June 8 (4.4%)	June 35 (8.2%)	June 43 (7.1%)	June 8 (9.3%)	June 35 (6.7%)
	July 18 (3.0%)	July 3 (1.6%)	July 15 (3.5%)	July 18 (3.0%)	July 2 (2.3%)	July 16 (3.1%)
	August 14 (2.3%)	August 2 (1.1%)	August 12 (2.8%)	August 14 (2.3%)	August 2 (2.3%)	August 12 (2.3%)
	September 14 (2.3%)	September 3 (1.6%)	September 11 (2.6%)	September 14 (2.3%)	September 3 (3.5%)	September 11 (2.1%)
	October 2 (0.3%)	October 0 (0%)	October 2 (0.5%)	October 2 (0.3%)	October 0 (0%)	October 2 (0.4%)
	November 3 (0.5%)	November 1 (0.5%)	November 2 (0.5%)	November 3 (0.5%)	November 0 (0%)	November 3 (0.6%)
	December 160 (26.3%)	December 54 (29.7%)	December 106 (24.8%)	December 160 (26.3%)	December 30 (34.9%)	December 130 (24.9%)
Triage Level at Admission	I 4 (0.7%)	I 0 (0%)	I 4 (0.9%)	I 7 (1.1%)	I 3 (3.5%)	I 4 (0.8%)
	II 133 (21.8%)	II 55 (30.2%)	II 78 (18.3%)	II 99 (16.3%)	II 31(36.0%)	II 68 (13.0%)
	III 336 (55.2%)	III 88 (48.4%)	III 248 (58.1%)	III 343 (56.3%)	III 43 (50.0%)	III 300 (57.4%)
	IV 107 (17.6%)	IV 32 (17.6%)	IV 75 (17.6%)	IV 144 (23.6%)	IV 9 (10.5%)	IV 135 (25.8%)
	V 29 (4.8%)	V 7 (3.8%)	V 22 (5.2%)	V 16 (2.6%)	V 0 (0.0%)	V 16 (3.1%)
Night admission (22 to 6 h) to the ED	94 (15.4%)	37 (20.3%)	57 (13.3%)	100 (16.4%)	18 (20.9%)	82 (15.7%)
Time of the day at admission	Evening and night (16:00 to 23:59 h) 240 (39.4%)	Evening and night (16:00 to 23:59 h) 70 (38.5%)	Evening and night (16:00 to 23:59 h) 170 (39.8%)	Evening and night (16:00 to 23:59 h) 211 (34.6%)	Evening and night (16:00 to 23:59 h) 32 (37.2%)	Evening and night (16:00 to 23:59 h) 179 (34.2%)
	Morning and afternoon (08:00 h to 15.59 h) 295 (48.4%)	Morning and afternoon (08:00 h to 15.59 h) 86 (47.3%)	Morning and afternoon (08:00 h to 15.59 h) 209 (48.9%)	Morning and afternoon (08:00 h to 15.59 h) 312 (51.2%)	Morning and afternoon (08:00 h to 15.59 h) 37 (43.0%)	Morning and afternoon (08:00 h to 15.59 h) 275 (52.6%)
	Early morning (00:00 to 07:59 h) 74 (12.2%)	Early morning (00:00 to 07:59 h) 26 (14.3%)	Early morning (00:00 to 07:59 h) 48 (11.2%)	Early morning (00:00 to 07:59 h) 86 (14.1%)	Early morning (00:00 to 07:59 h) 17 (19.8%)	Early morning (00:00 to 07:59 h) 69 (13.2%)
Lives near the hospital	390 (64%)	126 (69.2%)	264 (61.8%)	310 (50.9%)	49 (57.0%)	261 (49.9%)
History of Any Alcohol-Related Problem	182 (29.9%)			86 (14.1%)		
Report of Psychiatric Comorbidity	245 (40.2%)	91 (50.0%)	154 (36.1%)	112 (18.4%)	23 (26.7%)	89 (17.0%)
Type of Psychiatric Comorbidity	Psychotic Disorder 45 (18.4%)	Psychotic Disorder 17 (18.7%)	Psychotic Disorder 28 (18.2%)	Psychotic Disorder 14 (12.5%)	Psychotic Disorder 4 (17.4%)	Psychotic Disorder 10 (11.2%)
	Affective Disorder 70 (28.6%)	Affective Disorder 19 (20.9%)	Affective Disorder 51 (33.1%)	Affective Disorder 44 (39.3%)	Affective Disorder 8 (34.8%)	Affective Disorder 36 (40.4%)
	Personality Disorder 46 (18.8%)	Personality Disorder 25 (27.5%)	Personality Disorder 21 (13.7%)	Personality Disorder 14 (12.5%)	Personality Disorder 4 (17.4%)	Personality Disorder 10 (11.2%)
	Anxiety Disorder 47 (19.2%)	Anxiety Disorder 16 (17.6%)	Anxiety Disorder 31 (20.1%)	Anxiety Disorder 20 (17.9%)	Anxiety Disorder 5 (21.7%)	Anxiety Disorder 15 (16.9%)
	Others 37 (15.1%)	Others 14 (15.4%)	Others 23 (14.9%)	Others 20 (17.9%)	Others 2 (8.7%)	Others 18 (20.2%)
Report of other drug use-related disorders	276 (45.3%)	134 (73.6%)	142 (33.3%)	153 (25.1%)	46 (53.5%)	107 (20.5%)
Types of other drug use	Tobacco Use Disorders 169 (61.2%)	Tobacco Use Disorders 77 (57.5%)	Tobacco Use Disorders 92 (64.8%)	Tobacco Use Disorders 122 (79.7%)	Tobacco Use Disorders 36 (78.3%)	Tobacco Use Disorders 86 (80.4%)
	Cannabis Use Disorders 16 (5.8%)	Cannabis Use Disorders 3 (2.2%)	Cannabis Use Disorders 13 (9.2%)	Cannabis Use Disorders 12 (7.8%)	Cannabis Use Disorders 4 (8.7%)	Cannabis Use Disorders 8 (7.5%)
	Cocaine Use Disorders 17 (6.2%)	Cocaine Use Disorders 12 (9.0%)	Cocaine Use Disorders 5 (3.5%)	Benzodiazepine Use Disorders 6 (3.9%)	Benzodiazepine Use Disorders 1 (2.2%)	Benzodiazepine Use Disorders 5 (4.7%)
	Benzodiazepine Use Disorders 14 (5.1%)	Benzodiazepine Use Disorders 6 (4.5%)	Benzodiazepine Use Disorders 8 (5.6%)	Amphetamine Use Disorders 2 (1.3%)	Polysubstance Use Disorders 5 (10.9%)	Amphetamine Use Disorders 2 (1.9%)
	Amphetamine Use Disorders 6 (2.2%)	Amphetamine Use Disorders 3 (2.2%)	Amphetamine Use Disorders 3 (2.1%)	Opioid Use Disorders 1 (0.7%)		Opioid Use Disorders 1 (0.9%)
	Polysubstance Use Disorders 51 (18.5%)	Polysubstance Use Disorders 32 (23.9%)	Polysubstance Use Disorders 19 (13.4%)	Polysubstance Use Disorders 10 (6.5%)		Polysubstance Use Disorders 5 (4.7%)
	Others 3 (1.1%)	Others 1 (0.7%)	Others 2 (1.4%)			
Report of Organic Comorbidity	470 (77.2%)	144 (79.1%)	326 (76.3%)	378 (62.1%)	57 (66.3%)	321 (61.4%)

Data are reported as mean (M) with standard deviation (SD) for continuous variables or percentages (%) with counts for categorical variables unless otherwise specified. Lower levels of Triage imply higher acuity/clinical severity. ED, Emergency Department; ED FUs, Emergency Department Frequent Users; ARPs, Alcohol-Related Problems. Study period: from 1 December 2018 to 30 November 2019.

**Table 2:** Professional interventions received in the ED by ED FUs (cases) vs. non-frequent users (controls): descriptive statistics.

	ED FUs (*n* = 609)	ED FUs with ARPs (*n* = 182)	ED FUs without ARPs (*n* = 427)	Controls (*n* = 609)	Controls with ARPs (*n* = 86)	Controls without ARPs (*n* = 523)
Length of Stay (minutes)	M = 519.68 SD = 615.72	M = 601.85 SD = 595.729	M = 495.04 SD = 619.512	M = 377.56 SD = 460.81	M = 644.99 SD = 715.107	M = 333.59 SD = 387.742
Ambulance Arrival	148 (24.3%)	57 (31.3%)	91 (21.3%)	97 (15.9%)	30 (34.9%)	67 (12.8%)
Type of specialty that discharged the patient from the ED	Surgical 124 (20.4%)	Psychiatry 42 (23.1%)	Psychiatry 63 (14.8%)	Surgical 136 (22.3%)	Psychiatry 5 (5.8%)	Psychiatry 25 (4.8%)
	Trauma 40 (6.6%)	Internal Medicine 104 (57.1%)	Internal Medicine 236 (55.3%)	Trauma 105 (17.2%)	Internal Medicine 53 (61.6%)	Internal Medicine 285 (54.5%)
	Internal Medicine 340 (55.8%)	Trauma 12 (6.6%)	Trauma 28 (6.6%)	Internal Medicine 338 (55.5%)	Trauma 20 (23.3%)	Trauma 85 (16.3%)
	Psychiatry 105 (17.2%)	Surgical 24 (13.2%)	Surgical 100 (23.4%)	Psychiatry 30 (4.9%)	Surgical 8 (9.3%)	Surgical 128 (24.5%)
Situation at Discharge	Discharge home 500 (82.1%)	Discharge home 144 (79.1%)	Discharge home 356 (83.4%)	Discharge home 508 (83.4%)	Discharge home 56 (65.1%)	Discharge home 452 (86.4%)
	Hospital Admission 86 (14.1%)	Hospital Admission 29 (15.9%)	Hospital Admission 57 (13.3%)	Hospital Admission 81 (13.3%)	Hospital Admission 24 (27.9%)	Hospital Admission 57 (10.9%)
	Transfer to another center 23 (3.8%)	Transfer to another center 9 (4.9%)	Transfer to another center 14 (3.3%)	Transfer to another center 20 (3.3%)	Transfer to another center 6 (7.0%)	Transfer to another center 14 (2.7%)
Report of Alcohol Drinking Pattern	50 (8.2%)	40 (22.0%)	10 (2.3%)	30 (4.9%)	21 (24.4%)	9 (1.7%)
Psychiatric Assessment in ED	123 (20.2%)	56 (30.8%)	67 (15.7%)	38 (6.2%)	10 (11.6%)	28 (5.4%)
Social Assessment in ED	13 (2.1%)	11 (6.0%)	2 (0.5%)	4 (0.7%)	0 (0.0%)	4 (0.8%)
Assessment by a non-psychiatric medical specialty in ED	179 (29.4%)	57 (21.3%)	122 (28.6%)	142 (23.3%)	39 (45.3%)	103 (19.7%)

Data are reported as mean (M) with standard deviation (SD) for continuous variables or percentages (%) with counts for categorical variables unless otherwise specified. ED, Emergency Department; ED FUs, Emergency Department Frequent Users; ARPs, Alcohol-Related Problems. Study period: from 1 December 2018 to 30 November 2019.

**Table 3:** Detailed healthcare costs per a single ED visit ED FUs (cases) vs. non-frequent users (controls): descriptive statistics.

	ED FUs (*n* = 609)	ED FUs with ARPs (*n* = 182)	ED FUs without ARPs (*n* = 427)	Controls (*n* = 609)	Controls with ARPs (*n* = 86)	Controls without ARPs (*n* = 523)
Human Resources Costs (€)	M = 197.29 SD = 236.57	M = 241.86 SD = 246.51	M = 178.29 SD = 229.88	M = 141.31 SD = 163.32	M = 233.97 SD = 230.72	M = 126.08 SD = 144.06
Costs of Diagnostic Tests (€)	M = 15.47 SD = 28.45	M = 17.55 SD = 30.13	M = 14.58 SD = 27.69	M = 31.22 SD = 221.29	M = 33.10 SD = 65.73	M = 30.91 SD = 237.35
Blood bank costs (€)	M = 2.15 SD = 20.77	M = 2.57 SD = 24.5	M = 1.98 SD = 18.99	M = 0.82 SD = 12.12	M = 3.55 SD = 24.15	M = 0.38 SD = 8.64
Pharmacy Costs (€)	M = 6.37 SD = 5.575	M = 6.70 SD = 4.87	M = 6.24 SD = 6.09	M = 5.92 SD = 7.10	M = 7.68 SD = 6.67	M = 5.63 SD = 7.14
Medical supplies costs (€)	M = 5.85 SD = 5.93	M = 5.79 SD = 4.39	M = 5.87 SD = 6.48	M = 6.53 SD = 8.14	M = 7.36 SD = 7.22	M = 6.40 SD = 8.28
Other Costs (€)	M = 0.65 SD = 0.42	M = 0.67 SD = 0.29	M = 0.65 SD = 0.47	M = 0.63 SD = 0.59	M = 0.74 SD = 0.51	M = 0.61 SD = 0.59
Structural Costs (€)	M = 17.08 SD = 18.75	M = 20.64 SD = 19.23	M = 15.57 SD = 18.37	M = 13.98 SD = 21.64	M = 21.48 SD = 18.54	M = 12.75 SD = 21.88
Total Costs (€)	M = 244.87 SD = 268.86	M = 295.77 SD = 275.66	M = 223.17 SD = 263.25	M = 200.43 SD = 310.12	M = 307.88 SD = 265.70	M = 182.76 SD = 315.55

Data are reported as mean (M) with standard deviation (SD) for continuous variables or percentages (%) with counts for categorical variables unless otherwise specified. ED, Emergency Department; ED FUs, Emergency Department Frequent Users; ARPs, Alcohol-Related Problems. Study period: from 1 December 2018 to 30 November 2019.

### 3.2 Bivariate analyses

During the comparison of cases and controls in bivariate analyses, the mean total healthcare direct costs per ED visit were higher for FUs compared with non-frequent users [mean difference, 44.44 euros; 95% CI 13.4–75.5; t(608) = 2.811; *p* = 0.005]. The mean healthcare human resources costs for ED FU visits were higher by 55.97€ (95% CI 33.39–78.56) compared to costs for non-frequent users [t(608) = 4.868; *p* < 0.0005]. Mean LoS was longer for FUs by 142.117 minutes (95% CI 81.55–202.68) compared to non-frequent users [t (608) = 4.608, *p* = 0.0005].

In the bivariate analysis comparing ED FUs with and without ARPs and controls with and without ARPs, the mean total costs, healthcare human resources costs, and structural costs were higher, and the mean LoS was longer in the ARP groups from both FUs and non-frequent ED users (Table 4).

**Table 4:** Independent samples *t*-tests comparing ED FUs with and without ARP and non-frequent users of the ED with and without ARP.

	ED FUs with ARP and ED FUs without ARP			Controls with ARP and controls without ARP		
	Mean difference	95% CI of the difference	*p*-value	Mean difference	95% CI of the difference	*p*-value
Mean Total Costs (€)	**72.61**	**25.24–119.97**	0.003*	**125.13**	**62.28–187.98**	<0.0005*
Mean Healthcare Human Resources Costs (€)	**63.56**	**21.48–105.65**	0.003*	**107.89**	**56.95–158.84**	<0.0005*
Mean Structural Costs (€)	**5.07**	**1.76–8.37**	0.003*	**8.73**	**4.34–13.12**	<0.0005*
Mean Diagnostic Test Costs (€)	2.97	(−2.15)-8.10	0.255	2.19	(−48.43)-52.80	0.932
Mean Blood Bank Costs (€)	0.59	(−3.02)-4.20	0.749	3.17	(−2.06)-8.40	0.231
Mean Pharmacy Costs (€)	0.46	(−0.54)-1.46	0.362	**2.05**	**0.43–3.67**	0.013*
Mean Medical Supplies Costs (€)	(−0.08)	(−1.11)-0.95	0.879	0.96	(−0.89)-2.82	0.310
Mean Other Costs (€)	0.03	(−0.46)-0.10	0.461	0.13	(−0.003)-0.264	0.055
LoS (minutes)	**134.71**	**28.12–241.30**	0.013*	**311.40**	**154.63–468.18**	<0.0005*

ED FUs: Emergency Department Frequent Users; LoS: Length of Stay; ARP: Alcohol-Related Problems; CI: Confidence Interval; * and bold text indicate statistically significant results (*p* < 0.05).

### 3.3 Multivariate analyses

In a multiple linear regression model (Table 5) analyzing total costs per ED visit among FUs, we found that LoS, triage level, ambulance arrival, and the specialty that discharged the patient were significant predictors of direct costs. Longer LoS and ambulance arrival predicted higher costs per ED visit among FUs.

**Table 5:** Multiple linear regression models examining patient characteristics and professional interventions that influence direct total costs per ED visit among ED FUs.

	Unadjusted			Adjusted		
	B (95% CI)	*t*	*p*-value	B (95% CI)	*t*	*p*-value
Constant				112.837 (68.704, 156.969)	5.021	<0.0005
Age	3.070 (1.525, 4.614)	3.903	<0.0005			
LoS	0.397 (0.382, 0.411)	53.414	<0.0005	0.385 (0.370, 0.400)	50.256	<0.0005
Triage Level at Admission	−101.324 (−127.541, (−75.106))	−7.590	<0.0005	−19.427 (−31.355, (−7.500))	−3.199	0.001
Night admission	67.121 (8.093, 126.150)	2.233	0.026			
Ambulance arrival	138.693 (90.007, 187.379)	5.595	<0.0005	27.732 (3.008, 46.456)	2.236	0.026
Type of specialty that discharged the patient from the ED	−44.208 (−65.752, (−22.665))	−4.030	<0.0005	−11.401 (−20.661, (−2.142))	−2.418	0.016
History of any ARP	72.606 (26.186, 119.026)	3.072	0.002			
Other drug use-related disorders	43.272 (0.395, 86.150)	1.982	0.048			
Organic comorbidities	96.053 (45.610, 146.496)	3.740	<0.0005			
Situation at discharge	229.593 (190.423, 268.764)	11.511	<0.0005			
Report of alcohol drinking pattern	101.597 (24.015, 179.178)	2.572	0.010			
Assessed by a non-psychiatric medical specialty	212.888 (169.054, 256.723)	9.538	<0.0005			

Final adjusted Model: Adjusted R2 = 83.1%; F (4,604) = 747.716; *p* < 0.0005. ED, Emergency Department; ED FUs, Emergency Department Frequent Users; LoS, Length of Stay; ARP, Alcohol- Related Problem; CI, Confidence Interval. For Triage, Level I was the reference category. Lower levels of Triage imply higher acuity/clinical severity. For the Type of specialty that discharged the patient from the ED, “Psychiatry” was the reference category. For Situation at discharge, “Discharge home” was the reference category.

Among non-frequent ED users (Table 6), the significant predictors identified in multivariate analysis were LoS, triage level, ambulance arrival, specialty that discharged the patient, and receiving assessment by a non-psychiatric medical specialty in the ED. The longer duration of LoS, ambulance arrival, and receiving assessment by a non-psychiatric medical specialty in the ED predicted higher costs per ED visit.

**Table 6:** Multiple linear regression models examining patient characteristics and professional interventions that influence direct total costs per ED visit among non-frequent ED users.

	Unadjusted			Adjusted		
	B (95% CI)	*t*	*p*-value	B (95% CI)	*t*	*p*-value
Constant				292.286 (178.830, 405.742)	5.059	<0.0005
Age	4.282 (2.510, 6.053)	4.747	<0.0005			
Gender	58.574 (8.928, 108.221)	2.317	0.021			
LoS	0.371 (0.326, 0.416)	16.280	<0.0005	0.282 (0.233, 0.332)	11.165	<0.0005
Triage Level at Admission	−146.885 (−178.413,(−115.357))	−9.149	<0.0005	−62.225 (−92.265, (−32.186))	−4.068	<0.0005
Ambulance arrival	250.625 (186.150, 315.099)	7.634	<0.0005	80.516 (21.143, 139.890)	2.663	0.008
Type of specialty that discharged the patient from the ED	−51.001 (−78.487, (−23.515))	−3.644	<0.0005	−24.160 (−46.780, (−1.541))	−2.098	0.036
History of any ARP	125.129 (54.906, 195.352)	3.499	0.001			
Other drug use-related disorders	172.113 (116.841, 227.385)	6.115	<0.0005			
Organic comorbidities	101.925 (51.673, 152.178)	3.983	<0.0005			
Situation at discharge	240.283 (191.847, 288.718)	9.743	<0.0005			
Report of alcohol drinking pattern	284.538 (172.680, 396.396)	4.996	<0.0005			
Assessment by a non-psychiatric medical specialty in ED	262.160 (207.612, 316.707)	9.439	<0.0005	84.785 (32.426, 137.144)	3.180	0.002

Final adjusted Model: Adjusted R2 = 35.7%; F (5,603) = 68.509; *p* < 0.0005. ED, Emergency Department; LoS, Length of Stay; ARP, Alcohol-Related Problem; CI, Confidence Interval. For Triage, Level I was the reference category. Lower levels of Triage imply higher acuity/clinical severity. For the Type of specialty that discharged the patient from the ED, “Psychiatry” was the reference category. For situation at discharge, “Discharge home” was the reference category.

## 4 Discussion

To our knowledge, this investigation is one of the few to focus on understanding the influence of ARPs on healthcare costs related to frequent use of hospital emergency services in a public, universal, free-of-charge national health system.

In this secondary analysis, the mean direct total healthcare costs of a single ED visit in a general hospital were 22.2% more expensive among ED FUs compared to controls. It is clear that ED FUs present for emergency services much more frequently than do other patients and that, as a result, the total healthcare costs of this frequent use will be greater over time, as reported previously. A study conducted in the United States showed that, after a year, the global costs of attendance to emergency services were $10,465,216.07 among ED FUs compared with $1,012,610.21 among non-frequent ED users. However, past studies also pointed out that healthcare costs were similar for each ED visit between ED FUs and other users (29). Our results suggest that healthcare costs associated with ED frequent use are higher not only because of frequency but also because each consultation entails greater complexity, as indicated by the 37.6% longer mean LoS and 39.6% higher mean healthcare human resources costs for ED FUs compared with controls.

Results of bivariate analyses suggest that a history of ARPs was associated with an increase in mean total direct costs per visit among all ED users, raising mean human resources costs and LoS to an even greater degree among non-frequent users compared with FUs. Among ED FUs, a history of ARPs was linked to a 32.5% higher mean direct total cost per visit. Compared to ED FUs without ARPs, FUs with ARPs exhibited a 21.6% longer mean LoS, and each ED visit was 35.7% more expensive in terms of human resources costs. Among non-frequent users, ARPs were associated with a 68.5% higher mean direct total cost per visit. Compared with controls without ARPs, non-frequent users with ARPs had a mean LoS that was 93.3% longer, and each ED visit was 85.6% more expensive in terms of human resources costs. These results that suggest increased healthcare costs for patients with ARPs are similar to previously reported findings. In a Canadian sample of people with chronic diseases, psychiatric illnesses significantly increased the use of healthcare resources and their associated costs, whereas alcohol use disorders and other addictions had the highest rates of presentation to emergency services (15). In an Australian study involving ED patients, those who need extensive specialized treatment for alcoholism and other addictions attended the ED more frequently and incurred higher costs per visit. In addition, their hospital stays were usually longer (16). In a Catalan sample of adult primary healthcare patients, alcohol consumption was associated with increased charges from the public medical care system, showing a positive dose–response relationship (17).

The mean total direct cost per ED visit among ED FUs with ARPs was 295.77€ (SD = 275.66) in our analysis. Previous research on how ARPs affect frequent ED use–related costs is scarce, but estimates reported so far of healthcare costs for alcohol use–related ED presentations are generally higher. For instance, among ED users in the Netherlands, the average total expenses per patient seeking treatment for acute alcohol intoxication at the ED amounted to €1070 (encompassing estimated costs for ambulance transportation, ED visits, and hospital admission) (30). In an investigation involving adult ED users of a Belgian high-complexity hospital during a single year who attended the ED due to inebriety, the average estimated treatment cost was €541.32 per patient (31). However, most available estimates of urgent care costs related to alcohol use are reported from different countries, with distinct health systems and different costs of living.

In multivariate analyses, LoS, triage level, ambulance arrival, and type of specialty that discharged the patient were associated with total direct costs per ED visit among ED FUs in our sample. These factors were also associated with total direct costs among non-frequent users, as was undergoing assessment by a non-psychiatric medical specialty in the ED. According to these results, the most robust predictors of healthcare direct costs per ED visit would be the LoS, triage level, ambulance arrival, and discharge type of specialty for all users. The findings of the LoS and the triage level as predictors of ED healthcare costs are consistent with previous literature (32). Patients arriving by ambulance to the ED were noted to experience extended stays in the emergency services and incurred higher average expenses (33). The type of specialty that discharges the patient is also a predictor of ED healthcare costs, which is congruous with earlier evidence, as different types of diseases have been associated with diverse healthcare costs (34). Undergoing assessment by a non-psychiatric medical specialty is a predictor of costs among non-frequent users and is also consistent with previous knowledge. Multimorbidity is associated with an increased use of healthcare services, with associated costs (35). Nonetheless, given that ARPs were likely underreported in the reviewed charts, these results may underestimate emergency department costs attributable to ARPs.

### 4.1 Limitations

Some limitations of the current investigation should be noted. The first involves the case–control design, as retrospective data provide limited-quality evidence (28). Also, due to the focus of the original study on the relationship between substance use disorders and frequent ED use, clinical complexity was more finely assessed for substance use disorders and other psychiatric comorbidities and not so much for organic (non-psychiatric) medical comorbidities. Researchers were not blind to the study objectives or to the status of study participants (28). Since this is a single-center study, our findings might not be applicable to different contexts. Furthermore, variables of interest for this study could have been underreported in the charts. The literature suggests that psychiatric disorders (36), and especially addictions (25), are underreported in emergency medical reports. Therefore, it is possible that the findings underestimate the ED costs attributable to ARPs. Also, the estimates reflect only direct medical costs incurred only in the ED of one hospital, and the costs of using other resources of the healthcare system were not studied.

Despite these constraints, the academic background of abstractors equipped them with the expertise needed to precisely comprehend the subtleties of clinical data in the charts they reviewed. Additionally, they received standardized training in variable extraction and attended regular meetings with senior researchers to address disputes and review coding rules. Although prospective longitudinal studies provide higher-quality evidence, the clinical and social complexity of ED frequent users often makes long-term follow-up challenging. Retrospective designs are a useful initial approach for exploring large samples of this kind of patient.

Above all, the current study contributes to the limited body of research investigating expenses associated with frequent ED visits.

## 5 Conclusion

In a high-complexity public hospital, each FU attended the ED between 5 and 42 times per year, with an average of around 7 ED visits per patient. The mean direct total healthcare costs of a single ED visit were 22.2% higher among ED FUs than among matched, non-frequent users of emergency services. Given the association between a history of ARPs and a significantly increased likelihood of repeated emergency service use within the same sample, we propose that implementing targeted intervention protocols in the emergency room to address these issues simultaneously could mitigate the high healthcare costs associated with frequent ED use.

### Prior presentations

Some of the data included in this manuscript was previously presented as a short oral communication under the title “El precio de la hiperfrecuentación de urgencias hospitalarias: ¿qué papel juegan el uso de alcohol y otras drogas?” in the 4th International Congress-XLIX Jornadas Nacionales Socidrogalcohol, which was held in Tenerife, Spain, between 6^th^ and 8^th^ October 2022, and as a short oral communication under the title “Influence of alcohol use and other addictive disorders in the costs of Emergency Department Frequent Use” in Lisbon Addictions 2022 (European Conference on Addictive Behaviors and Dependencies), which was held in Lisbon, Portugal, between 23rd and 25^th^ November 2022.

## Data Availability

The original contributions presented in the study are included in the article/Supplementary materials, further inquiries can be directed to the corresponding author.
