# Supplementary material for: Effects of alcohol-related problems on the costs of frequent emergency department use: an economic analysis of a case–control study in Spain
Source: Front Public Health. 2024 Dec 3;12:1322327. doi: 10.3389/fpubh.2024.1322327 (PMC11653189; doi:10.3389/fpubh.2024.1322327)
Supplement: Supplementary file 2 [file Table_2.pdf]

Supplementary Material 2.

Diagnostic codes according to the ICD-10 and DSM-5 included in the definition of "history of any alcohol-related problem".

**ICD-10**	**DSM-5**
category F10 (Alcohol-related disorders) and the corresponding subsections	F10.129, F10.229 or F10.929 (Alcohol Intoxication)
T51.0-X and subsections (Toxic effect of ethanol)	F10.10 and F10.20 (Alcohol Use Disorder)
G31.2 (degeneration of the nervous system due to alcohol)	
G72.1 (alcoholic myopathy)	
I42.6 (alcoholic cardiomyopathy)	
K29.2 (alcoholic gastritis)	
K70 (alcoholic hepatopathy) and subsections	
K85.2 (alcohol-induced acute pancreatitis) and subsections	
K86. 0 (chronic alcohol-induced pancreatitis)	
R78.0 (finding / presence of alcohol in the blood)	
Y90 (evidence of alcohol involvement determined by blood alcohol level) and subsections	
Z71.4 and Z71.41 (Assessment and Counselling of Alcohol abuse)	
