# Supplementary material for: Effects of alcohol-related problems on the costs of frequent emergency department use: an economic analysis of a case–control study in Spain
Source: Front Public Health. 2024 Dec 3;12:1322327. doi: 10.3389/fpubh.2024.1322327 (PMC11653189; doi:10.3389/fpubh.2024.1322327)
Supplement: Supplementary file 3 [file Table_3.pdf]

Supplementary Material 3.

Detailed methodology to compute economic costs

Healthcare human resources costs

Healthcare human resources costs are the costs of nursing, medical, administration and general services personnel incurred during the patient's visit.

Several variables are taken into account to calculate healthcare human resources costs: salary per minute according to the professional category, Length of Stay in the ED, the number of staff members and boxes per floor, the floor from which the patient was discharged (this ED is located in a specific building of the hospital, the ED building, and different acuity levels are distributed to different floors) and the percentage of dedication of the professional.

- **Rate per minute:** the salary of the collective labour agreement is taken into account according to the professional category. It also includes the proportional part of vacations and planned absenteeism. In Spain, most Residency Programs last four years, some of them five.

Professional category	Rate/minute
Administration staff	0.44 €
Nursing assistant	0.35 €
Social workers	0.55 €
Nurse	0.55 €
Healthcare assistant	0.35 €
Fourth-fifth year resident doctor	0.32 €
Medical Specialist	0.97 €
Cleaning staff	0.30 €

- **Floor's Staff composition:** the average number of each professional category by floor is taken into account. The professional categories included are:

ED Floor	Administration staff	Nursing assistant	Social Workers	Nurse	Healthcare assistant	Fourth-fifth year resident doctor	Medical Specialist	Cleaning Staff
**BPARO**	1	1		3	1		1	1
**PUR2**	0.5	1		3	0.75	3	3	0.75
**PUR2- IV and V**	0.5	1		1	0.25	0	2	0.25
**PUR3**	1	2		4	1	4	3	1
**PUR4**	1	2		4	1	3	2	1
**PUR6**		1		1	1	1	1	1

**HEL2**	1	3		3	1	1	2	1
**PUR1 - Admissions**	2		1		1			

- **Provision of boxes per floor:** the number of emergency boxes on each floor is taken into account.

Floor	Boxes
BPARO	5
PUR2	16
PUR2- IV and V	8
PUR3	18
PUR4	22
PUR6	6
HEL2	28

- **Floor from which the patient was discharged:** the staffing varies depending on the ground where the patient receives treatment.

- **Length of Stay in the ED:** In minutes. From the moment the patients enter the ED to the moment they leave it (either because they are discharged home, admitted to an inpatient unit in the same hospital or transferred to another centre).

- **Percentage of dedication of the professional:** refers to the dedication that the professional has towards the patient. For this calculation, the number of boxes and the number of professionals of the same professional category that are in each floor are taken into account.

Percentage of dedication of the professional: Number of professionals in the same categories in the ED floor / Number of boxes in the ED floor

Example: If floor 2 has a total of 10 boxes and has 2 nurses, the dedication of the nurse to the patient is 2/10=0.2.

Calculation of healthcare human resources costs: Length of stay in the discharge floor * Minute rate per discharge floor

Minute rate per discharge floor: (Administration staff rate per minute x percentage of dedication administration staff in the floor) + (Nursing assistant rate per minute x percentage of dedication nursing assistant in the floor) + (social workers' rate per minute x percentage of dedication of social workers in the floor) + (nurse's rate per minute x percentage of nurse's dedication in the floor) + (healthcare assistant's rate per minute + percentage of healthcare assistant's dedication in the floor) + (fourth-fifth year resident doctor rate per minute x percentage of fourth-fifth year

residency doctor's dedication in the floor) + (Medical Specialist rate per minute x percentage of Medical Specialist's dedication in the floor) + (cleaning staff rate per minute x percentage of Cleaning Staff's dedication in the floor)

PUR2	PUR2 - IV-V	PUR3	PUR4	PUR6	HEL2	BPARO
0,42 €	0,42 €	0,47 €	0,33 €	0,48 €	0,23 €	0,83 €

Sometimes patients are initially admitted to one floor of the ED but their clinical situation changes so they are transferred to a different floor of the ED building to finalize their stay. If the patients are initially admitted to one floor of the ED but end up their stay in the ED in a different floor, then both the costs of the admission floor and the discharge floor are taken into account.

Calculation of healthcare human resources costs: (Length of stay in the admission ED floor + minute rate per admission floor) + (Length of stay in the discharge floor * Minute rate per discharge floor)

Diagnostics tests' Costs

For the economic calculation of the diagnostic tests of the Diagnostic Imaging Center (DIC) the internal rates of the hospital are used. The DIC diagnostic tests are assigned to the patient.

Costs of medical supplies, Pharmacy costs and other costs

For the calculation of medical supplies' costs, Pharmacy and other costs, the expense of these items of the cost center that refers to the floor are divided by the total number of discharges from that floor. The cost of these items is not assigned to the individual patient, so for their calculation the average cost per patient per ED floor must be obtained.
